# Ferroptosis and Metabolic Dysregulation: Emerging Chemical Targets in Cancer and Infection

**DOI:** 10.3390/molecules30143020

**Published:** 2025-07-18

**Authors:** Marta Pawłowska, Jarosław Nuszkiewicz, Dorian Julian Jarek, Alina Woźniak

**Affiliations:** 1 Department of Medical Biology and Biochemistry, Faculty of Medicine, Ludwik Rydygier Collegium Medicum in Bydgoszcz, Nicolaus Copernicus University in Toruń, 24 Karłowicza St., 85-092 Bydgoszcz, Poland; jnuszkiewicz@cm.umk.pl; 2 Student Research Club of Medical Biology and Biochemistry, Department of Medical Biology and Biochemistry, Faculty of Medicine, Ludwik Rydygier Collegium Medicum in Bydgoszcz, Nicolaus Copernicus University in Toruń, 24 Karłowicza St., 85-092 Bydgoszcz, Poland; 309897@stud.umk.pl

**Keywords:** ACSL4, ferroptosis, GPX4, iron metabolism, lipid peroxidation, LOX enzymes, metabolomics, PUFA, redox regulation

## Abstract

The distinctive nature of ferroptosis is that it is induced chemically and signifies a regulated cell death dependent on iron-dependent lipid peroxidation. The mechanism of ferroptosis involves oxidative damage to the membrane lipids. It differs from apoptosis and necroptosis, triggering metabolic changes in the iron-lipid homeostasis and antioxidant defense, such as glutathione (GSH) and glutathione peroxidase 4 (GPX4). Herein, the molecular mechanisms of ferroptosis and its role in the tumorigenesis process and infection-related diseases are presented. It also discusses metabolic reprogramming as a factor that modifies the levels of cell-sensitizing polyunsaturated fatty acids (PUFAs), iron dysregulation, and oxidative stress in aggressive cancers and inflammatory diseases such as sepsis, tuberculosis, and COVID-19. Particular attention is given to chemical modulators of ferroptosis, including synthetic inducers and inhibitors, as well as bioactive natural compounds. Our focus is on the significance of analytical tools, such as lipidomics and metabolomics, in understanding the phenomenon of ferroptosis. Finally, we explore novel therapeutic approaches targeting ferroptosis in cancer and infectious diseases, while navigating both the opportunities and challenges in drug development. The review then draws on chemical biology and disease pathology to propose promising areas of study for ferroptosis-related therapies.

## 1. Introduction

### 1.1. Ferroptosis: Definition and Molecular Hallmarks

Ferroptosis is a form of regulated cell death that depends on iron for its occurrence and is characterized by the lethal accumulation of lipid peroxides in cellular membranes [1]. The molecular background of ferroptosis differs from that of other forms of cell death. The basis of ferroptosis is the peroxidation of polyunsaturated fatty acids (PUFAs) in membrane phospholipids [2]. PUFAs undergo iron-catalyzed oxidation, often aided by enzymes such as lipoxygenases, which leads to the formation of toxic lipid peroxides and irreversible damage to the cell membrane [3]. At the same time, the accumulation of divalent iron (Fe^2+^) drives the Fenton reaction, generating reactive oxygen species (ROS), which further enhance lipid peroxidation. The critical role of iron is indicated by the fact that its chelation effectively blocks ferroptosis [4].

Another characteristic feature of ferroptosis is the disruption of antioxidant defenses, particularly the glutathione (GSH)/glutathione peroxidase 4 (GPX4) system [5]. GSH depletion disrupts the cellular redox balance, while loss of GPX4 activity stops the reduction of lipid peroxides, allowing uncontrolled peroxidation and subsequent cell death [6,7]. This biochemical mechanism distinguishes ferroptosis from other regulated models of cell death. Apoptosis is a caspase-dependent process in which DNA is broken down and the cell is disassembled in an orderly manner, leaving the cell membrane intact. In contrast, ferroptosis does not involve caspases and is driven by iron-dependent oxidative stress (OS). Ferroptosis does not lead to DNA fragmentation and the controlled cell disintegration seen in apoptosis [8,9]. Necroptosis involves the signaling pathways of receptor-interacting protein kinase 1 (RIPK1), receptor-interacting protein kinase 3 (RIPK3), and mixed lineage kinase domain-like protein (MLKL), ultimately leading to the rupture of the plasma membrane. In comparison, ferroptosis occurs independently of these kinases [8,10]. Pyroptosis, on the other hand, is a type of cell death that causes inflammation, triggered by proteins such as gasdermin and caspase-1, which create pores in the cell membrane [11]. In contrast, ferroptosis occurs due to lipid damage within the cell, rather than the degradation of proteins [12].

What makes ferroptosis chemical is its basis in redox biology, which is governed by three interdependent principles. Iron acts as a catalyst for the ROS generation. PUFAs serve as substrates for lipid peroxidation. Protective systems such as GPX4 are unable to detoxify lipid peroxides [13]. Together, these elements form a self-amplifying loop of oxidative membrane damage, distinguishing ferroptosis from more signal-driven forms of cell death. This chemically driven nature not only defines its mechanism but also presents therapeutic opportunities in diseases like cancer and neurodegeneration, where modulating lipid peroxidation and redox balance may influence cell fate [14].

### 1.2. Overview: Relevance of Ferroptosis in Cancer and Infection

Ferroptosis has emerged as an essential mechanism in disease pathogenesis, with a significant role in cancer progression and infection. Because ferroptosis stands out from other forms of regulated cell death, it presents novel therapeutic targets [15].

In cancer, ferroptosis has two crucial roles. Tumor suppressor pathways, like the p53 pathway, help trigger ferroptosis to destroy cancer cells [16]. However, cancer cells can develop mechanisms to avoid ferroptosis. This makes standard treatments less effective. This is a serious problem in cancers that do not respond well to therapy. Modulating the sensitivity of cancer cells to ferroptosis by reducing GSH levels or increasing lipid peroxide levels may increase the efficacy of treatment and promote cancer cell death [17,18].

Ferroptosis also plays a significant role in the course of infection. In severe cases, COVID-19 contributes to tissue damage and hyperinflammation, which causes exacerbation of lung and systemic organ failure. Disruption of iron metabolism and lipid peroxidation in infected cells intensifies cytokine storms, which lead to a worsening prognosis [19,20,21]. In sepsis, bacteria can induce ferroptosis in immune cells such as macrophages. The host’s defense mechanisms are weakened. This leads to uncontrolled infection and ultimately damage to many organs. It is worth noting that the incidence of ferroptosis strongly correlates with mortality rates due to sepsis [22]. In the case of *Mycobacterium tuberculosis* infection, bacteria manipulate the host’s iron metabolism to inhibit ferroptosis, which helps the pathogens survive [23]. On the other hand, therapeutic induction of ferroptosis in infected macrophages could potentially result in increased bacterial clearance. This is therefore an essential point of intervention [24].

Metabolic dysregulation further influences ferroptosis sensitivity. Disruption of iron homeostasis, lipid metabolism, and antioxidant defense creates an environment conducive to ferroptosis [13]. The increased availability of PUFAs, combined with a weakened ability to neutralize lipid peroxides, increases the susceptibility of cells to damage [25].

Together, these findings indicate that ferroptosis is a key biological process in the study of modern diseases. Targeting this process may have significant therapeutic potential, including in the treatment of cancer and infectious diseases.

### 1.3. Aims of the Review

This paper aims to provide a comprehensive and up-to-date review of the molecular mechanisms underlying ferroptosis, with particular emphasis on its chemical basis. It also aims to explain how metabolic perturbations render cells susceptible to ferroptosis. Furthermore, the review highlights the influence of chemical modulators on ferroptosis, discussing their therapeutic potential as well as the challenges associated with them. Finally, the review aims to outline current and future therapeutic strategies for treating neoplastic diseases and infections that target ferroptosis. The novel aspect of this paper is its simultaneous focus on chemistry and metabolism. It connects how ferroptosis works in both cancer and infectious diseases. This dual focus is uncommon in current research.

## 2. Chemical Basis of Ferroptosis

### 2.1. Lipid Peroxidation Pathways

Ferroptosis is a unique form of regulated cell death characterized by the iron-dependent accumulation of lipid peroxides in cellular membranes [26]. This process begins and spreads through the peroxidation of PUFAs, mainly arachidonic acid (AA) and adrenic acid (AdA), which are highly prone to oxidation because of their bis-allylic hydrogen atoms [27]. The sensitivity of PUFAs to peroxidation makes their presence within membrane phospholipids a critical determinant of ferroptosis susceptibility. Therefore, understanding the chemical and enzymatic mechanisms that drive PUFA peroxidation is central to elucidating the basis of ferroptosis [28].

Lipid peroxidation can proceed via two main routes: non-enzymatic, free radical-driven autoxidation and enzymatic oxidation catalyzed by specific oxygenases. The non-enzymatic pathway is initiated by hydroxyl radicals (^•^OH), which are primarily generated through the Fenton reaction involving ferrous iron (Fe^2+^) and hydrogen peroxide (H_2_O_2_) [29]. Once initiated, lipid radicals (L^•^) propagate chain reactions leading to the formation of lipid hydroperoxides (PUFA-OOH). This autocatalytic process is particularly destructive in the absence of sufficient antioxidant defenses, such as GPX4, which generally reduces PUFA-OOH to non-toxic lipid alcohols (PUFA-OH) [30].

The enzymatic component of lipid peroxidation in ferroptosis primarily involves lipoxygenases (LOXs), a family of non-heme iron-containing dioxygenases [15]. Among them, arachidonate 15-lipoxygenase (ALOX15) has been most strongly implicated in ferroptotic cell death due to its ability to catalyze the formation of hydroperoxy derivatives of membrane-bound PUFAs [31]. Additionally, the coordinated activity of acyl-CoA synthetase long-chain family member 4 (ACSL4) and lysophosphatidylcholine acyltransferase 3 (LPCAT3) plays a critical role in incorporating oxidizable PUFAs into phospholipid bilayers, thereby creating substrates for LOX-mediated peroxidation. This process, termed PUFA-phospholipid remodeling, amplifies ferroptotic signaling by enriching membranes with oxidizable lipids [32].

In addition to their role in initiating lipid peroxidation, free radicals such as hydroxyl and peroxyl species contribute to the formation of highly reactive aldehyde by-products, including malondialdehyde (MDA) and 4-hydroxynonenal (4-HNE) [33]. These secondary lipid peroxidation products are not only markers of oxidative damage but also bioactive molecules that can form adducts with proteins, DNA, and phospholipids, further exacerbating cellular dysfunction [34]. In the context of ferroptosis, such by-products may act as amplifiers of damage, promoting a self-reinforcing cycle of membrane destabilization and OS [35].

Significantly, the susceptibility to lipid peroxidation is tightly regulated by the lipid composition of cellular membranes. Enrichment in PUFA-containing phospholipids, particularly phosphatidylethanolamines (PEs), has been shown to be a prerequisite for ferroptotic death [36]. ACSL4 and LPCAT3 not only promote the incorporation of PUFA but also determine the specific molecular species available for peroxidation. This remodeling process is highly dynamic and can be influenced by cellular metabolic state, oncogenic signaling, and inflammatory cues, highlighting the intersection of ferroptosis with broader biological processes [37].

Recent studies have also implicated mitochondria in regulating lipid peroxidation during ferroptosis [38]. Although the initiation of ferroptosis does not strictly depend on mitochondrial function, mitochondrial metabolism can modulate the redox environment by producing ROS through the electron transport chain, especially under conditions of iron overload [38]. Moreover, mitochondria contain cardiolipin, a PUFA-rich phospholipid, which can be oxidized and contribute to the pool of lipid peroxides [39]. While the precise role of mitochondria remains context-dependent, their involvement adds a layer of complexity to the spatial regulation of ferroptosis.

Given the central role of lipid peroxidation in the execution of ferroptosis, cells have evolved specialized antioxidant systems to counteract this process [40]. Chief among them is the GSH-dependent activity of GPX4, which selectively reduces phospholipid hydroperoxides (PL-OOH) to their corresponding alcohols (PL-OH), thus interrupting the lipid peroxidation chain reaction [41]. The inactivation or depletion of GPX4, either through genetic suppression, pharmacological inhibition, or GSH depletion, removes this critical defense, tipping the redox balance toward lethal accumulation of lipid peroxides [42].

In parallel, cells also utilize radical-trapping antioxidants (RTAs), such as vitamin E (α-tocopherol), coenzyme Q10 (ubiquinol), and synthetic molecules like ferrostatin-1 and liproxstatin-1, to neutralize lipid radicals and terminate chain-propagating reactions [43,44]. The presence or absence of these protective agents strongly influences ferroptosis sensitivity. It is increasingly recognized as a determinant of selective vulnerability in different cell types, including cancer cells and immune cells during infection [45].

In summary, lipid peroxidation serves as both a biochemical hallmark and a mechanistic driver of ferroptosis. It results from a tightly regulated interplay between membrane lipid composition, iron-dependent oxidative chemistry, and enzymatic remodeling pathways. The vulnerability of cells to ferroptosis is therefore dictated not only by their antioxidant capacity but also by the availability and localization of oxidizable lipid species. Understanding these molecular events provides a foundation for targeted chemical intervention, whether by promoting ferroptosis in therapy-resistant cancer or mitigating ferroptotic damage in infectious and inflammatory contexts.

### 2.2. Iron Metabolism and ROS Generation

Iron is a central player in the ferroptotic process, acting as both a cofactor and a catalyst in the generation of ROS [46]. Within the cellular milieu, iron exists in dynamic equilibrium between Fe^3+^ and Fe^2+^ states, enabling it to participate in redox reactions that drive oxidative damage [36]. Among iron-mediated redox reactions, the Fenton reaction plays a central role in generating ^•^OH, which initiates lipid peroxidation cascades and ultimately leads to ferroptotic cell death [47].

The intracellular availability of catalytically active iron is governed by the labile iron pool (LIP), a redox-active reservoir of loosely bound Fe^2+^ and Fe^3+^ [48]. The size and reactivity of this pool are tightly controlled through a network of proteins involved in iron uptake, storage, and export [48]. Key among them are transferrin receptor 1 (TfR1), which mediates cellular iron import; ferritin, which stores excess iron in a redox-inert form; and ferroportin, the sole known iron exporter [49]. Disruption of this homeostatic network, whether through increased iron uptake or impaired sequestration, results in an expanded LIP and enhances susceptibility to ferroptosis [50].

In pathological contexts such as cancer and infection, iron metabolism is frequently dysregulated in a manner that favors oxidative stress and ferroptotic vulnerability [36]. Many cancer cells exhibit a phenotype known as “iron addiction”, characterized by upregulation of TfR1 and downregulation of ferritin and ferroportin, effectively expanding the intracellular iron pool to support rapid proliferation and metabolic activity [51]. Similarly, during infection and inflammation, immune cells may alter iron trafficking to restrict microbial access to iron, a defense mechanism known as nutritional immunity. However, this can paradoxically lead to local iron overload and increased ROS production, thereby promoting ferroptotic signaling in host or pathogen-targeted cells [52].

One of the key mechanisms by which cells mobilize stored iron into the LIP is ferritinophagy, a selective autophagic process that degrades ferritin. This process is mediated by the nuclear receptor coactivator 4 (NCOA4), which binds to ferritin heavy chain (FTH1) and facilitates its delivery to lysosomes for degradation [53]. The resulting release of free Fe^2+^ from ferritin dramatically increases the redox-active iron pool, thereby enhancing ROS generation and sensitizing cells to ferroptosis. Upregulation of NCOA4-dependent ferritinophagy has been documented in both tumor cells and during certain infections, underscoring its role as a pro-ferroptotic mechanism in stress conditions [54].

In addition to NCOA4, several other regulators modulate iron homeostasis and contribute to ferroptotic sensitivity. For instance, iron regulatory proteins (IRP1 and IRP2) post-transcriptionally control the expression of key iron metabolism genes by binding to iron-responsive elements (IREs) in target mRNAs. Under iron-deficient conditions, IRPs stabilize the mRNAs of TfR1 and divalent metal transporter 1 (DMT1), while repressing translation of ferritin and ferroportin, effectively increasing intracellular iron uptake and retention [55]. Dysregulation of this axis, whether through oncogenic signaling, inflammatory cytokines, or hypoxia, can tip the balance toward iron overload and potentiate ferroptosis [45].

The intersection of iron metabolism with ferroptosis has also opened avenues for therapeutic exploitation. Iron chelators such as deferoxamine (DFO) and deferiprone (DFP) can suppress ferroptotic death by sequestering free Fe^2+^ and limiting the Fenton reaction [56]. Conversely, pharmacological activation of ferritinophagy or inhibition of ferritin expression may sensitize resistant cancer cells to ferroptosis inducers. This duality underscores the importance of context: while iron mobilization can promote cell death in tumors, it must be mitigated in tissues undergoing inflammatory or infection-related injury to prevent collateral damage [57].

In summary, iron metabolism lies at the heart of ferroptosis, both as a generator of pro-oxidant species and as a modifiable determinant of cellular redox status. The LIP serves as a catalytic hub for ROS generation via the Fenton reaction, while upstream regulatory mechanisms, including transferrin-mediated uptake, ferritin storage, and NCOA4-driven ferritinophagy, orchestrate the availability of redox-active iron. These pathways are highly responsive to metabolic, oncogenic, and inflammatory stimuli, rendering ferroptosis a context-dependent outcome of systemic and local iron dynamics. Importantly, the ferroptotic process cannot proceed solely based on iron accumulation; it also requires the failure of protective antioxidant systems to counteract lipid peroxidation. Thus, iron overload acts synergistically with glutathione depletion and GPX4 inactivation to drive cells beyond the threshold of oxidative damage.

### 2.3. Glutathione and GPX4 System

While the GSH-GPX4 axis is the canonical suppressor of ferroptosis, cells also possess auxiliary antioxidant pathways that can partially compensate for GPX4 loss [58]. One such system involves ferroptosis suppressor protein 1 (FSP1), formerly known as apoptosis-inducing factor mitochondria-associated 2 (AIFM2). FSP1 functions independently of GSH and GPX4 by regenerating coenzyme Q10 (CoQ10) and its reduced form CoQ10H_2_ (ubiquinol), a lipophilic antioxidant that can trap lipid radicals within membranes [59]. This process is driven by nicotinamide adenine dinucleotide phosphate (NADPH) as an electron donor and plays a critical role in inhibiting lipid peroxidation at the plasma membrane. The FSP1–CoQ10 axis has been shown to confer resistance to ferroptosis in a variety of cell types and represents an essential target in therapy-resistant cancers [14].

Regulation of GPX4 expression and activity occurs at multiple levels. At the transcriptional level, factors such as NRF2 (nuclear factor erythroid 2–related factor 2) upregulate GPX4 and genes involved in GSH biosynthesis under oxidative stress [60]. Post-transcriptionally, GPX4 mRNA is subject to regulation by RNA-binding proteins and microRNAs, while its enzymatic activity depends on the availability of the rare amino acid selenocysteine. The incorporation of selenocysteine into GPX4 requires the specialized selenocysteine insertion sequence (SECIS) element and adequate dietary selenium, making GPX4 uniquely sensitive to micronutrient availability and translational regulation [61].

The metabolic status of the cell further influences ferroptosis susceptibility through its impact on redox balance. NADPH, generated primarily via the pentose phosphate pathway (PPP), is essential for maintaining reduced GSH levels via glutathione reductase [62]. Inhibition of NADPH-producing pathways or excessive ROS burden can tip the balance toward GSH oxidation (to oxidized glutathione, glutathione disulfide (GSSG), impairing GPX4 activity [63]. Moreover, metabolic rewiring in cancer cells, including increased glutaminolysis and altered mitochondrial function, can either support antioxidant defenses or, conversely, enhance ROS generation, depending on context and oncogenic drivers [64].

Taken together, the GSH-GPX4 system acts as the central redox checkpoint that determines whether lipid peroxidation remains under control or progresses to ferroptotic cell death. Its unique capacity to detoxify membrane-bound lipid hydroperoxides distinguishes it from other antioxidant systems and underscores its critical role in ferroptosis suppression [65]. Importantly, GPX4 activity is intimately linked to broader metabolic circuits, including amino acid transport, selenium availability, and NADPH-dependent redox cycling, providing multiple metabolic entry points for chemical modulation [41].

The functional interplay between GPX4 and alternative antioxidant systems such as FSP1-CoQ10 emphasizes the redundancy and adaptability of ferroptosis resistance mechanisms [66]. This layered defense architecture enables certain cells, particularly therapy-resistant cancer phenotypes, to evade ferroptotic death even in the face of oxidative stress. Consequently, pharmacological strategies that simultaneously impair GSH synthesis, inhibit GPX4, and block auxiliary antioxidant pathways are being explored to overcome this resistance [67].

**Figure 1:**
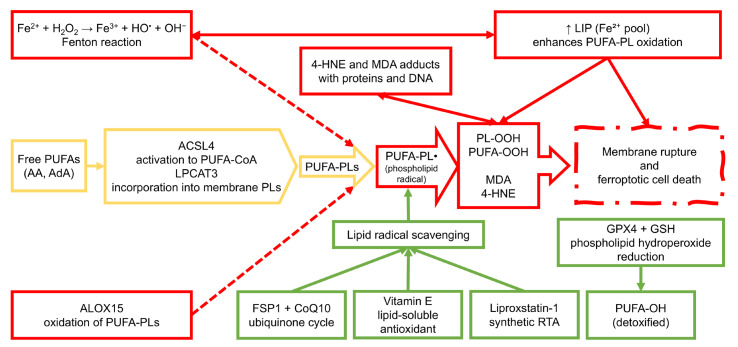
Lipid peroxidation cascade and regulatory pathways in ferroptosis. Free polyunsaturated fatty acids (PUFAs), such as arachidonic acid (AA) and adrenic acid (AdA), are activated by acyl-CoA synthetase long-chain family member 4 (ACSL4) and incorporated into membrane phospholipids (PLs) via lysophosphatidylcholine acyltransferase 3 (LPCAT3), forming PUFA-containing PLs (PUFA-PLs). These are oxidized by lipoxygenases (e.g., ALOX15) or non-enzymatically via hydroxyl radicals (•OH) generated through the Fenton reaction involving ferrous iron (Fe^2+^), producing phospholipid radicals (PUFA-PL•) and lipid hydroperoxides (PL-OOH, PUFA-OOH). Toxic by-products such as malondialdehyde (MDA) and 4-hydroxynonenal (4-HNE) cause membrane rupture and ferroptotic cell death. Cells counteract ferroptosis using antioxidant defenses. Glutathione peroxidase 4 (GPX4), in the presence of glutathione (GSH), reduces lipid hydroperoxides to non-toxic PUFA alcohols (PUFA-OH). Additional protection is provided by radical-trapping antioxidants (RTAs), including vitamin E, ferrostatin-1, and liproxstatin-1, as well as ferroptosis suppressor protein 1 (FSP1), which regenerates reduced coenzyme Q10 (CoQ10) via the ubiquinone cycle. Abbreviations used: AA—arachidonic acid; ACSL4—acyl-CoA synthetase long-chain family member 4; AdA—adrenic acid; ALOX15—arachidonate 15-lipoxygenase; CoQ10—coenzyme Q10; Fe^2+^—ferrous iron; FSP1—ferroptosis suppressor protein 1; GPX4—glutathione peroxidase 4; GSH—glutathione; LIP—labile iron pool; LPCAT3—lysophosphatidylcholine acyltransferase 3; MDA—malondialdehyde; PL—phospholipid; PL-OOH—phospholipid hydroperoxide; PUFA—polyunsaturated fatty acid; PUFA-OH—detoxified PUFA alcohol; PUFA-OOH—PUFA hydroperoxide; PUFA-PL—PUFA-containing phospholipid; PUFA-PL•—phospholipid radical; RTA—radical-trapping antioxidant; •OH—hydroxyl radical; 4-HNE—4-hydroxynonenal.

In the context of disease, the GSH-GPX4 axis represents a double-edged sword. While its activity protects normal tissues from oxidative injury during infection and inflammation, its overexpression in tumors may contribute to chemoresistance and immune evasion [68]. Understanding how this system is regulated and manipulated across different pathophysiological settings is therefore essential for designing targeted therapeutic interventions. A graphical summary of the lipid peroxidation cascade underlying ferroptosis is presented in Figure 1.

### 2.4. Iron-Independent Ferroptosis-like Mechanisms

While ferroptosis has traditionally been defined as an iron-dependent, lipid peroxidation-driven form of regulated cell death, recent evidence suggests the existence of ferroptosis-like processes that can proceed independently of classical Fe^2+^-Fenton chemistry [69]. One such mechanism involves dihydroorotate dehydrogenase (DHODH), a mitochondrial enzyme that supports ubiquinol regeneration and protects against mitochondrial lipid peroxidation. In cells lacking GPX4 activity, DHODH functions as a parallel antioxidant defense, particularly within the inner mitochondrial membrane, delaying or preventing ferroptosis [70].

Moreover, mitochondria-derived ROS can directly initiate lipid peroxidation even in the absence of increased labile iron, especially under conditions of impaired NADPH regeneration or CoQ10 depletion [71]. Additionally, non-GPX4 peroxidases and peroxisome-mediated lipid oxidation have been implicated in ferroptosis-like cell death under specific metabolic contexts [72].

These findings expand the traditional view of ferroptosis and suggest that lipid peroxidation itself may be the ultimate trigger of cell death in specific scenarios. Acknowledging these alternative pathways provides a more nuanced understanding of ferroptosis regulation and broadens the therapeutic landscape for diseases where iron-independent OS is prominent.

## 3. Chemical Modulators of Ferroptosis

### 3.1. Ferroptosis Inducers

Ferroptosis inducers are compounds that trigger an iron-dependent form of regulated cell death [73]. Ferroptosis inducers are gaining popularity due to their promise in treating cancer. Cancer cells often have high iron levels and active iron metabolism. This makes them more susceptible to ferroptosis [74].

Erastin was first discovered in 2003 as a genotype-selective antitumor agent and is a well-characterized inducer of ferroptosis (see Table 1) [73]. It blocks system Xc^−^, a cystine/glutamate antiporter responsible for importing cystine into cells. By inhibiting this transporter, erastin lowers intracellular cysteine levels [75]. Cysteine is a key compound for GSH synthesis. Without GSH, GPX4 loses its ability to neutralize harmful lipid peroxides, resulting in uncontrolled lipid oxidation and cell death [76]. Erastin also facilitates the uptake of chemotherapeutic drugs in resistant tumors by affecting iNOS activity [77].

Another compound, RAS-selective lethal 3 (RSL3), induces ferroptosis through a different mechanism than erastin. It does not interfere with cystine uptake or GSH synthesis. Instead, it directly binds to and inhibits GPX4. The function of the GPX4 enzyme is blocked by covalent modification of its active site (see Table 1). This causes the accumulation of toxic lipid peroxides. RSL3 is particularly effective in tumors with high GPX4 expression, such as non-small cell lung cancer [78,79].

FIN56 is a novel ferroptosis inducer that triggers ferroptosis through a dual mechanism (see Table 1). This leads to the degradation of GPX4 and a reduction in the cell’s antioxidant capacity. Additionally, FIN56 activates squalene synthase, an enzyme involved in the biosynthesis of cholesterol. This activation leads to the depletion of CoQ10, a lipid-soluble antioxidant, thereby increasing OS within the cell [80].

FINO_2_ is an organic peroxide containing a 1,2-dioxolane skeleton. This inducer of ferroptosis contains both an endoperoxide and a hydroxyl group in its structure, which is crucial for its activity (see Table 1) [81]. Unlike other ferroptosis inducers, it does not directly inhibit GPX4 or block cystine uptake. Instead, it indirectly disrupts GPX4 activity, which in turn leads to the oxidation of intracellular iron, resulting in the generation of ROS that drive lipid peroxidation [82]. Ferroptosis is selectively initiated in cancer cells by FINO_2_, as observed in the fibrosarcoma cell line HT-1080 and the B-lymphocytic leukemia cell line RS411. Furthermore, FINO_2_ is more effective against cancer cells than non-malignant cells of the same type [83].

In addition to known ferroptosis inducers, compounds such as sulfasalazine and sorafenib also play a significant role in inducing ferroptosis. Sulfasalazine, initially developed for its anti-inflammatory effects, is a combination of sulfapyridine and salicylate (see Table 1). Commonly prescribed for conditions such as rheumatoid arthritis and inflammatory bowel disease, it exhibits both immunomodulatory and anti-inflammatory properties. Although its precise mechanism of action has not been fully elucidated, research has revealed that sulfasalazine also displays potential anti-cancer properties [84]. Like erastin, sulfasalazine inhibits system Xc^−^, leading to GSH depletion [85]. Sorafenib is an orally administered, bioavailable inhibitor that targets multiple kinases and is currently utilized in the treatment of advanced cancers such as thyroid carcinoma, hepatocellular carcinoma, and renal cell carcinoma (see Table 1) [84]. It also disrupts the functioning of system Xc^−^, which contributes to its potential anticancer effects [86].

Ferroptosis inducers have been shown to exhibit cytotoxicity against multiple tumor cells, both in vivo and in vitro [73]. The described ferroptosis inducers affect different points of cellular antioxidant defense. System Xc^−^ inhibitors, such as erastin and sulfasalazine, reduce GSH levels in cells. Direct GPX4 inhibitors, such as RSL3, prevent the enzyme from functioning. GPX4 degraders, such as FIN56, lower the amount of GPX4 protein. Multi-acting agents such as FINO_2_ indirectly affect GPX4 and also enhance cellular iron oxidation. Despite their different mechanisms of action, all ferroptosis inducers effectively affect the accumulation of lipid peroxides. This makes them valuable tools used in cancer therapy.

### 3.2. Ferroptosis Inhibitors

Ferroptosis inhibitors work by blocking iron-dependent lipid peroxidation. Compared to ferroptosis inducers, the number of inhibitors is relatively small, and small-molecule compounds constitute only a small portion. Ferroptosis inhibitors can be used in cancer immunotherapy and other diseases, such as stroke [73]. These compounds use different mechanisms to protect cells. However, RTAs and iron chelators are two major therapeutic approaches [87].

Liproxstatin-1 and ferrostatin-1 are potent RTAs that stop the chain reactions associated with lipid peroxidation (see Table 2). Both compounds are arylamines, and numerous studies have demonstrated that arylamines exhibit effective radical-trapping antioxidant properties [88]. They are more effective in lipid membranes than in solution, allowing them to neutralize lipid peroxyl radicals more efficiently than natural antioxidants, such as vitamin E. The radical chain reaction can be stopped by these RTAs, leading to the formation of stable nonradical products and therefore preventing cell membrane disruption [89]. Liproxstatin-1 and ferrostatin-1 action prevent the spread of lipid damage without interfering with enzymes such as lipoxygenases. Additionally, they protect mitochondria from the accumulation of lipid peroxides and reduce inflammation by decreasing cytokines such as IL-6 and TNF-α [90,91].

Other lipophilic antioxidants, such as vitamin E and CoQ10, also prevent ferroptosis by neutralizing lipid radicals [92]. However, their protective effects are weaker than those of synthetic RTAs. For example, vitamin E reacts with peroxyl radicals more slowly than ferrostatin-1 in lipid membranes (see Table 2) [93]. CoQ10 plays a key role as a lipid-soluble antioxidant, protecting against ferroptosis. The FSP1-CoQ10-NADPH axis drives this protection (see Table 2). FSP1 regenerates CoQ10 using NADPH via cholesterol-driven enhancement of CoQ10 synthesis in the mevalonate pathway [94]. Clinically, CoQ10 helps prevent retinal degeneration and sensitizes cancer cells to ferroptosis inducers [95].

Iron chelators, such as DFO and DFP, bind free iron in the cell, thereby stopping the Fenton reaction (see Table 2) [96]. These chelators have been shown to block ferroptosis in vascular smooth muscle cells exposed to cigarette smoke [97]. In addition, these compounds restore GSH balance by reducing iron-induced OS and preventing mitochondrial fragmentation [98].

The therapeutic potential of ferroptosis inhibitors has been demonstrated in various diseases. For example, liproxstatin-1 and ferrostatin-1 can protect brain cells in the hippocampus from ferroptosis, suggesting a significant role in neuroprotection [99,100]. DFO has been shown to reduce aortic damage resulting from exposure to cigarette smoke [101]. This may therefore indicate a potential use of this compound in the treatment of cardiovascular diseases. Inhibition of ferroptosis is a promising strategy for the treatment of diseases associated with OS. It seems that the actions of RTAs and iron chelators can complement each other. By inhibiting cellular damage, they can improve patient outcomes.

### 3.3. Natural Compounds Targeting Ferroptosis

Polyphenols, terpenoids, and flavonoids are natural compounds that can potentially affect ferroptosis. These bioactive molecules interact with GPX4. Natural compounds could also regulate iron metabolism and affect lipid oxidation pathways [102]. The use of these compounds is helpful in the treatment of cancer, ischemia-reperfusion injury, as well as drug-induced toxicity [3,103].

Among polyphenols, quercetin promotes ferroptosis by reducing the expression of GPX4 and system Xc^−^ (SLC7A11), which leads to GSH depletion and increased lipid peroxidation (see Table 3). It also affects the increase in intracellular iron concentration through nuclear receptor coactivator 4 (NCOA4)-dependent ferritinophagy, thereby enhancing the production of reactive oxygen species [104]. Epigallocatechin gallate (EGCG) suppresses GPX4 activity and blocks NRF2-dependent antioxidant response, thereby making cancer cells more susceptible to ferroptosis [105]. In addition, EGCG reduces mitochondrial membrane potential and promotes the accumulation of lipid peroxides [106]. In turn, curcumin promotes ferroptosis by reducing SLC7A11 levels and activating ACSL4-dependent lipid remodeling. It also chelates iron, which supports Fenton reactions and enhances OS [107].

Terpenoids, including artemisinin derivatives, induce ferroptosis by using iron to activate endoperoxide bridges. They generate carbon-centered radicals that enhance lipid peroxidation [108]. Some terpenoids, such as parthenolide, lower GSH levels by inhibiting γ-glutamylcysteine ligase (GCLC), which further reduces GPX4 activity (see Table 3) [109,110].

Flavonoids, such as baicalein and luteolin, stimulate ferroptosis through two primary actions. They affect iron metabolism by increasing transferrin receptor 1 (TFR1) expression to boost iron uptake and by reducing ferritin levels. At the same time, they enhance lipid peroxidation by suppressing GPX4 and activating ACSL4, which promotes the incorporation of PUFAs into cell membranes, making them more prone to oxidative damage (see Table 3) [111,112,113]. Through these combined effects, flavonoids help overcome drug resistance in cancers such as colorectal and liver cancer and reduce tissue damage during ischemia-reperfusion injury by targeting the interconnected metabolic networks of iron regulation, lipid metabolism, and antioxidant defense [114].

### 3.4. Therapeutic Relevance of Ferroptosis-Modulating Compounds in Disease Contexts

Several ferroptosis-regulating compounds discussed in this review have been evaluated in disease-specific settings, highlighting their potential as therapeutic agents in cancer and infection.

In the context of cancer, sorafenib, approved initially for hepatocellular carcinoma (HCC), has been shown to induce ferroptosis by depleting intracellular glutathione and inactivating system xCT [115]. Studies have demonstrated that sorafenib-resistant HCC cells regain ferroptosis sensitivity when treated in combination with GPX4 inhibitors, suggesting a synergistic potential [116,117,118].

Erastin, a well-characterized system xCT inhibitor, promotes ferroptosis in glioma, leukemia, and colorectal cancer cell lines. In glioblastoma models, erastin sensitizes tumor cells to radiation and chemotherapeutics via iron-dependent lipid peroxidation [119]. Similarly, RSL3, a GPX4 inhibitor, enhances ferroptotic death in therapy-resistant triple-negative breast cancer (TNBC) and pancreatic ductal adenocarcinoma (PDAC) models [120,121].

Regarding infectious diseases, liproxstatin-1 and ferrostatin-1 have demonstrated protective effects in preclinical models of sepsis, where excessive ferroptosis contributes to immune cell dysfunction and multi-organ damage [122]. Liproxstatin-1 alleviated lipid peroxidation in macrophages and endothelial cells, reduced systemic cytokine levels, and improved survival in mouse models of endotoxemia [123].

Furthermore, artemisinin derivatives, primarily used as anti-malarial agents, have been reported to induce ferroptosis-like death in both cancer cells and specific pathogens by disrupting iron homeostasis and elevating ROS levels [124]. Sulfasalazine has shown ferroptosis-inducing activity in colorectal and lung cancers, offering additional translational potential [125].

These studies underscore the clinical relevance of ferroptosis-modulating agents and support further investigation into their application as targeted therapies for both malignant and infectious diseases.

## 4. Metabolic Dysregulation and Ferroptosis Sensitivity

### 4.1. Lipid Metabolism Reprogramming

Reprogramming of lipid metabolism is a hallmark of both cancer and infection, and it plays a decisive role in modulating cellular sensitivity to ferroptosis [126]. In cancer, oncogenic signaling pathways, including RAS, MYC, and PI3K-AKT, drive extensive remodeling of lipid biosynthetic and remodeling enzymes, resulting in elevated levels of PUFAs within membrane phospholipids [127]. As discussed in Section 2.1, the enzymatic incorporation of PUFAs into membrane phospholipids by ACSL4 and LPCAT3 establishes a biochemical basis for ferroptotic vulnerability. In cancer, upregulation of these enzymes in response to oncogenic signals has been linked to increased ferroptosis sensitivity and is being explored as a therapeutic target.

In the setting of infection, immune cells, particularly macrophages and neutrophils, undergo lipidomic reprogramming in response to inflammatory stimuli such as cytokines and pathogen-associated molecular patterns (PAMPs) [128]. This includes upregulation of lipid synthesis enzymes and remodeling of the membrane lipidome to favor inflammatory signaling and phagocytic function. Importantly, these changes often involve increased PUFA content, creating a pro-ferroptotic environment. For instance, lipopolysaccharide (LPS)-stimulated macrophages exhibit elevated ACSL4 expression and enhanced susceptibility to lipid peroxidation, suggesting that ferroptosis may contribute to immune-mediated tissue injury during severe infections [129].

The inflammatory microenvironment characteristic of both tumors and infections further influences lipid metabolism and ferroptosis susceptibility [130]. Pro-inflammatory cytokines, including tumor necrosis factor-α (TNF-α), interleukin-6 (IL-6), and interferon-γ (IFN-γ), have been shown to modulate the expression of key enzymes involved in PUFA metabolism [131]. These cytokines can enhance ACSL4 and LPCAT3 expression, upregulate ALOX isoforms, and downregulate antioxidant defenses-creating a biochemical milieu favoring lipid peroxidation [132]. The result is a feed-forward loop in which inflammation promotes oxidative stress and lipid remodeling, which in turn sensitizes cells to ferroptosis and amplifies tissue damage.

In cancer, these processes are further compounded by metabolic rewiring driven by the tumor’s demand for biomass and redox balance. Many cancer cells display increased uptake of exogenous fatty acids and upregulation of de novo lipogenesis, with a preference for PUFA synthesis and incorporation [133]. This reprogramming is not merely a byproduct of proliferation, but rather an adaptive mechanism that shapes ferroptosis sensitivity. Tumors with high PUFA content are more vulnerable to ferroptotic inducers, especially under conditions of antioxidant suppression or iron overload. This vulnerability is being actively explored as a therapeutic window in tumors refractory to conventional treatments [134].

Importantly, the cellular context dictates the outcome of lipid metabolic reprogramming. While tumor cells may be driven toward ferroptosis through PUFA enrichment, immune cells within the same microenvironment can undergo similar remodeling but respond differently [135]. For instance, activated macrophages and dendritic cells may resist ferroptosis by engaging alternative antioxidant programs, including NRF2-mediated transcriptional responses and FSP1-driven radical scavenging, which allow them to sustain function in oxidative environments. These differential responses underscore the need for targeted ferroptosis modulation that accounts for cell-type-specific metabolic programs [136].

In summary, lipid metabolism reprogramming represents a fundamental determinant of ferroptosis sensitivity in both cancer and infection. The enzymatic machinery that governs PUFAs activation and incorporation into phospholipids, notably ACSL4 and LPCAT3, is upregulated in response to oncogenic and inflammatory cues, resulting in membranes primed for peroxidation. This biochemical remodeling, when coupled with impaired antioxidant defense or elevated iron availability, sets the stage for ferroptotic cell death. The implications of this process are twofold. On one hand, it offers a potential therapeutic target: tumors enriched in PUFA-containing lipids may be selectively vulnerable to ferroptosis inducers. On the other hand, it poses a risk during infection or inflammation, where immune cell remodeling may inadvertently promote ferroptotic injury in host tissues. As such, understanding the context-specific drivers and modulators of lipidomic reprogramming is essential for safely harnessing ferroptosis as a therapeutic strategy.

### 4.2. Iron Dysregulation in Disease Context

Iron homeostasis is profoundly altered in cancer, where malignant cells frequently exhibit a phenotype of “iron addiction”. This metabolic adaptation supports the increased demand for DNA synthesis, mitochondrial respiration, and oxidative phosphorylation seen in proliferating tumor cells [62]. Key features of this phenotype include upregulation of TfR1, downregulation of ferroportin (FPN), and suppression of ferritin expression, all of which contribute to expansion of the LIP and heightened production of ROS [137].

As detailed in Section 2.2, ferritinophagy mediated by NCOA4 mobilizes stored iron and promotes ferroptosis by expanding the LIP. In cancer, this mechanism supports proliferation but simultaneously primes tumor cells for ferroptotic death, particularly under conditions of oxidative or inflammatory stress. Its dual role as both a growth enabler and a death trigger makes it an attractive target for therapeutic modulation [138].

Beyond supporting tumor growth, iron dysregulation also has therapeutic implications. Many ferroptosis-inducing agents, including GPX4 inhibitors and cystine transport blockers, are significantly more effective in iron-overloaded environments [36]. Conversely, tumors with low iron availability, either due to high ferritin expression or impaired ferritinophagy, may exhibit intrinsic resistance. These findings have spurred interest in combining iron modulators (e.g., ferritinophagy activators or iron supplementation) with ferroptosis inducers to overcome resistance and selectively target iron-addicted tumors [139].

In infectious and inflammatory diseases, iron metabolism is tightly regulated as part of the host’s defense strategy to limit microbial proliferation—a concept known as nutritional immunity [140]. Upon exposure to pro-inflammatory cytokines such as IL-6, the liver upregulates the iron-regulatory hormone hepcidin, which induces degradation of ferroportin, the only known cellular iron exporter. This leads to systemic hypoferremia and intracellular iron sequestration, particularly within macrophages, hepatocytes, and enterocytes [141]. While this response aims to deprive pathogens of access to extracellular iron, it also results in intracellular iron accumulation, particularly in immune cells.

This paradoxical sequestration creates a pro-oxidant microenvironment, particularly in inflamed tissues, where elevated labile iron can amplify oxidative stress through Fenton-type chemistry [35]. Activated macrophages, for example, can accumulate iron through increased transferrin receptor expression and phagocytosis of senescent red blood cells. In the presence of excessive ROS, whether from mitochondrial sources or NADPH oxidases, these iron-rich cells become susceptible to ferroptotic death [56]. This mechanism is believed to contribute to tissue injury in sepsis, tuberculosis, and viral infections, such as coronavirus disease 2019 (COVID-19), where both systemic inflammation and OS are prominent [142].

Moreover, some pathogens have evolved mechanisms to manipulate host iron handling in their favor, subverting nutritional immunity and inadvertently contributing to ferroptotic conditions. Gram-negative bacteria such as *Escherichia coli*, *Salmonella enterica*, and *Pseudomonas aeruginosa* produce high-affinity iron-chelating molecules known as siderophores (e.g., enterobactin, salmochelin, pyoverdine), which scavenge iron from host transferrin and lactoferrin [143]. This hijacking of extracellular iron pools can trigger compensatory hepcidin production and intracellular iron sequestration in host cells, paradoxically increasing labile iron and ROS in phagocytes and epithelial barriers [144].

*Mycobacterium tuberculosis*, the causative agent of tuberculosis, expresses siderophore-like molecules (mycobactin, carboxymycobactin) and modulates macrophage iron metabolism by inhibiting ferroportin expression and promoting ferritin accumulation. These alterations favor intracellular iron retention and oxidative imbalance, potentially priming infected macrophages for ferroptosis-like cell death [145].

In the context of viral infections, hepatitis C virus (HCV) and human immunodeficiency virus (HIV) have been shown to upregulate hepcidin and alter ferritin expression, leading to intracellular iron accumulation in hepatocytes and macrophages [146]. Similarly, severe acute respiratory syndrome coronavirus 2 (SARS-CoV-2), the agent responsible for COVID-19, has been associated with hyperferritinemia and dysregulated iron metabolism, particularly in severe cases. These perturbations may contribute to the death of lung epithelial cells via ferroptosis and to systemic organ damage during cytokine storm conditions [147].

Disruptions in iron metabolism, whether driven by oncogenic signals or inflammatory responses, act as potent amplifiers of ferroptosis susceptibility by expanding the LIP and intensifying oxidative pressure on cellular membranes [148]. In cancer, this dysregulation arises from metabolic adaptations that support proliferation but inadvertently prime tumor cells for ferroptotic death [149]. In contrast, infection-induced iron retention, while part of an antimicrobial defense, can have collateral effects that promote ferroptotic injury in host tissues [150].

What unifies these divergent pathological contexts is the convergence of iron overload and redox imbalance, which together constitute a biochemical trigger for the execution of lipid peroxidation and ferroptosis [151]. These findings underscore the importance of metabolic context in determining cellular fate, offering new opportunities for therapeutic intervention. Targeting iron-handling pathways may serve not only to sensitize tumors to ferroptosis inducers but also to protect vulnerable tissues during infections by restoring iron equilibrium and suppressing ferroptotic signaling.

### 4.3. Metabolic Ferroptosis Vulnerabilities Across Cancer Types

Recent studies have shown that susceptibility to ferroptosis varies significantly depending on the type and stage of cancer. The interplay of lipid metabolism, iron homeostasis, and antioxidant mechanisms defines specific susceptibilities to ferroptosis, creating opportunities for targeted therapeutic intervention [25,152].

In hepatocellular carcinoma (HCC), tumor cells exhibit increased expression of ACSL4 and transferrin receptor 1 (TfR1), as well as decreased GPX4 activity. This molecular profile increases sensitivity to lipid peroxidation [153,154]. Sorafenib, initially developed for HCC, was later shown to induce ferroptosis, particularly when combined with GPX4 inhibitors or xCT blockers [155,156]. This finding provides a strong rationale for ferroptosis-based combination therapies in liver cancer.

Glioblastoma multiforme (GBM) is characterized by extensive iron uptake and accumulation coupled with increased PUFAs content in cell membranes. These tumors also upregulate xCT activity in the cystine/glutamate antiporter system, contributing to redox imbalance [157,158]. Preclinical models have demonstrated that factors such as erastin and RSL3 sensitize GBM cells to ferroptosis [159]. Furthermore, inhibition of xCT or FSP1 in patient-derived xenografts further enhances ferroptotic cell death, suggesting promising therapeutic strategies for this condition [160].

Pancreatic ductal adenocarcinoma (PDAC), known for its highly resistant metabolic phenotype, is characterized by robust NADPH regeneration and glutathione synthesis. These tumors often exhibit xCT overexpression and impaired iron storage [161]. Targeting glutathione biosynthesis or inhibiting SLC7A11 significantly increases ferroptosis sensitivity in vitro and in mouse models, suggesting a novel vulnerability for this treatment-resistant tumor [162].

Triple-negative breast cancer (TNBC) subtypes often express high levels of NRF2 and FSP1, which protect against ferroptotic stress [163]. However, inhibition of FSP1 or pharmacological removal of coenzyme Q10 resensitizes TNBC cells to ferroptosis-inducing agents. Importantly, combining ferroptosis-inducing agents with immune checkpoint blockade has demonstrated synergistic effects in mouse models of TNBC [164,165].

These examples underline the importance of contextualizing ferroptosis modulation within the unique metabolic architecture of individual cancers. Integrating metabolic and disease-specific information will be essential for the successful clinical translation of ferroptosis-targeted therapies.

## 5. Ferroptosis in Disease Contexts

As introduced in Section 1.2, ferroptosis is implicated in both cancer and infectious diseases. This section explores these relationships in greater mechanistic and therapeutic detail.

### 5.1. Cancer

Ferroptosis plays a complex but critical role in cancer, functioning both as a natural tumor-suppressive mechanism and as a vulnerability that can be therapeutically exploited. When properly regulated, ferroptosis helps eliminate malignant cells, but when disrupted, it contributes to tumor growth, therapy resistance, and disease recurrence. This dual role makes ferroptosis an essential focus in cancer research and treatment development [26].

**Figure 2:**
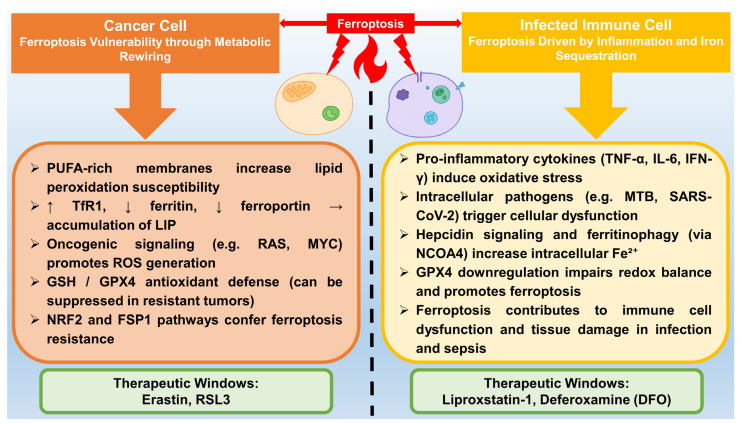
Distinct metabolic and inflammatory contexts modulate ferroptosis susceptibility in cancer and infection. In cancer cells, ferroptosis vulnerability results from metabolic rewiring, PUFA-enriched membranes, iron accumulation (↑ TfR1, ↓ ferritin and ferroportin), and increased ROS levels. However, antioxidant systems such as the GSH/GPX4 axis and protective signaling through NRF2 and FSP1 can confer resistance. In infected immune cells, inflammation-driven cytokines (e.g., TNF-α, IL-6, IFN-γ), pathogen-mediated stress, hepcidin signaling, and NCOA4-dependent ferritinophagy lead to iron overload and GPX4 suppression, thereby impairing redox balance and promoting ferroptosis. Disease-specific ferroptosis mechanisms are linked to distinct therapeutic windows. Cancer cells respond to ferroptosis inducers such as erastin and RSL3, while immune cells under inflammatory stress may benefit from ferroptosis inhibitors such as liproxstatin-1 and deferoxamine (DFO). Abbreviations used: DFO—deferoxamine, FSP1—ferroptosis suppressor protein 1, GPX4—glutathione peroxidase 4, GSH—glutathione, IFN-γ—interferon gamma, IL-6—interleukin 6, LIP—labile iron pool, MTB—Mycobacterium tuberculosis, NCOA4—nuclear receptor coactivator 4, NRF2—nuclear factor erythroid 2–related factor 2, PUFA—polyunsaturated fatty acids, RAS—rat sarcoma proto-oncogene, ROS—reactive oxygen species, RSL3—RAS-selective lethal 3, SARS-CoV-2—severe acute respiratory syndrome coronavirus 2, TfR1—transferrin receptor 1, TNF-α—tumor necrosis factor alpha.

One key pathway in ferroptosis-mediated tumor suppression involves the tumor suppressor protein p53. Activation of p53 promotes ferroptosis through ALOX12, a lipoxygenase that oxygenates polyunsaturated fatty acids to produce lipid peroxides. This pathway operates independently of GPX4 and ACSL4 and has been demonstrated to suppress tumor formation, as the loss or mutation of ALOX12 accelerates cancer development in lymphoma models [166,167]. Notably, p53 triggers ferroptosis without directly inhibiting GPX4. Instead, it relies on increased OS to induce cell death in cancer cells (see Figure 2) [168].

However, many cancer cells develop resistance to ferroptosis, allowing them to survive under conditions that would usually be lethal [169]. One central resistance mechanism involves the activation of NRF2, a transcription factor stabilized by mutations in KEAP1—mutations that are particularly common in non-small cell lung cancer [170]. Stabilized NRF2 boosts the expression of antioxidant genes such as SLC7A11 and GPX4, which neutralize lipid peroxides. It also promotes iron storage, lowering the levels of free iron needed to drive Fenton reactions and lipid oxidation [171]. Another resistance pathway involves FSP1, which reduces CoQ10 into a potent lipid-soluble antioxidant. This process blocks lipid peroxidation independently of GPX4 and is mainly active in KEAP1-mutant cancers, where FSP1 expression is elevated due to NRF2 activity [172]. Additionally, epigenetic changes can influence resistance to treatment. For instance, in sepsis-related liver injury, loss of EZH1 reduces repressive histone marks on the NRF2 gene promoter, further increasing NRF2 expression and strengthening ferroptosis resistance [173].

To counter these resistance mechanisms, combination therapies that induce ferroptosis in conjunction with standard treatments are being explored. In neuroblastoma, combining auranofin—a thioredoxin reductase inhibitor—with chemotherapy enhances cell death by promoting ferritin degradation and iron overload, effectively eliminating chemoresistant cells [174]. However, not all combinations are beneficial. For example, using the GPX4 inhibitor RSL3 with etoposide may backfire, as RSL3 can trigger a compensatory increase in GPX4 that dampens the overall treatment effect [175].

In some cases, ferroptosis can be used to target residual cancer cells that survive initial treatments. In triple-negative breast cancer, residual tumor cells after chemotherapy show elevated iron levels, making them more vulnerable to ferroptosis [176,177,178]. Combining RSL3 with FSP1 inhibitors has been shown to eliminate these cells and delay tumor recurrence in patient-derived xenograft (PDX) models [179,180]. Advanced drug delivery methods, such as those using nanoparticles, can improve treatment outcomes. These nanoparticles deliver both iron ions and chemotherapeutic agents, such as hydroxycamptothecin, triggering simultaneous apoptosis and ferroptosis for enhanced tumor killing [181,182].

The clinical potential of targeting ferroptosis is significant. By inhibiting resistance pathways such as NRF2 and FSP1 and integrating ferroptosis inducers into existing cancer therapies, researchers can open new avenues for treating stubborn cancers. For instance, dual inhibition of FSP1 and NRF2 can sensitize KEAP1-mutant non-small cell lung cancers to ferroptosis-induced cell death [183]. In hepatocellular carcinoma, blocking miR-141-3p restores the effectiveness of sorafenib by suppressing the Keap1-Nrf2 axis [184]. The findings described above suggest that ferroptosis is a promising therapeutic target for the development of cancer treatment strategies.

### 5.2. Infection

Ferroptosis plays a multifaceted role in infections. This form of cell death contributes to both host defense and harmful tissue damage in bacterial and viral infections, mainly through impaired iron metabolism and increased lipid peroxidation (see Figure 2) [185,186].

In bacterial infections, ferroptosis is particularly important in conditions such as sepsis and tuberculosis [186]. In polymicrobial sepsis, immune cells undergo ferroptosis due to the suppression of GPX4. Proinflammatory signals, such as lipopolysaccharide (LPS) and TNF-α, reduce GPX4 activity. This allows lipid peroxides to accumulate, triggering an immunogenic form of cell death. This not only amplifies inflammation but also causes organ damage through acyl-CoA synthetase ACSL4-dependent membrane remodeling [22,187]. The kidneys and liver are particularly susceptible to this type of damage. The use of ferroptosis inhibitors such as liproxstatin-1 in animal models has been shown to reduce tissue damage and improve survival [188]. In tuberculosis, pathogens manipulate host iron metabolism by increasing hepcidin levels, which retain iron in macrophages and promote a pro-ferroptotic state. Infected macrophages exhibit elevated levels of free iron and lipid-based ROS, along with GPX4 degradation. The suppression of ferroptosis in this context has been shown to reduce bacterial burden, suggesting that this cell death pathway also contributes to microbial control [52,189].

Viral infections also show a strong association with ferroptosis, especially in severe cases of COVID-19 and chronic hepatitis B and C [190,191]. In the case of COVID-19, lung epithelial cells undergo ferroptosis due to iron overload. During the disease, high ferritin levels, impaired hepcidin regulation, and inactivation of GPX4 by the SARS-CoV-2 ORF3a protein are observed [76]. Post-mortem samples show lipid peroxidation, a marker of which is an increased level of 4-hydroxynonenal (4-HNE), a marker of lipid damage [192]. Ferroptosis in the case of COVID-19 exacerbates cytokine storms, blood clotting issues, and, consequently, increased patient mortality [193]. In the case of hepatitis B and C virus infections, the chronic presence of viral infection promotes ferroptosis in liver cells through other mechanisms. Hepatitis C virus (HCV) core protein suppresses the cystine transporter SLC7A11 by depleting GSH, whereas hepatitis B virus (HBV) X protein increases ACSL4 expression, promoting PUFAs peroxidation. Chronic infection also leads to hepatic iron accumulation, which accelerates fibrosis through ferroptotic mechanisms [194,195,196].

Iron overload is a common feature of many infections. It is a key trigger for ferroptosis. Pathogens often disrupt iron balance by stimulating the production of hepcidin, which limits iron export and retention in cells. The accumulation of reactive iron drives the Fenton reaction, generating ROS and increasing oxidative damage. At the same time, infections deplete cellular antioxidants such as GPX4 and GSH. This makes cells sensitive to ferroptosis [194]. These mechanisms can increase host tissue damage. On the other hand, they can also limit pathogen growth by reducing the availability of nutrients. In cases such as sepsis and tuberculosis, where excessive ferroptosis leads to harmful inflammation and organ failure, the use of ferroptosis inhibitors, such as ferrostatins, may protect tissues [186]. In intracellular infections, such as salmonellosis, inducing ferroptosis with agents like erastin may help eliminate pathogens by killing infected cells [189]. New treatment approaches are being investigated, including the use of iron chelators in viral hepatitis and NRF2 activators in COVID-19. The goal of these therapeutic strategies is to restore iron balance and reduce collateral damage [197].

The role of ferroptosis in infectious diseases remains context-dependent and, at times, paradoxical. While ferroptosis contributes to pathogen clearance in some intracellular infections, such as *Salmonella* and *M. tuberculosis*, it also exacerbates tissue damage and immunosuppression during systemic inflammation, particularly in sepsis and severe COVID-19. These contradictory findings reflect the complexity of ferroptotic signaling in immune cells and underline the need for cell-type-specific and stage-specific therapeutic approaches [186,198]. Further in vivo studies are essential to clarify when ferroptosis acts as a protective versus detrimental process in infectious pathology.

### 5.3. Neurodegenerative Diseases

Ferroptosis has recently been linked to the progression of several neurodegenerative diseases, including Alzheimer’s disease (AD), Parkinson’s disease (PD), and amyotrophic lateral sclerosis (ALS). Increased OS, mitochondrial dysfunction, and iron accumulation in specific brain regions are characteristic of these conditions [199]. In PD, dopaminergic neurons in the substantia nigra accumulate iron and undergo lipid peroxidation [200]. In AD, abnormal lipid metabolism and reduced antioxidant defenses promote the susceptibility of hippocampal neurons to ferroptosis [201]. GPX4 deficiency and GSH depletion have also been observed in neurodegenerative models [202].

Notably, several ferroptosis inhibitors have demonstrated neuroprotective effects in preclinical studies. Compounds such as ferrostatin-1 and liproxstatin-1 have been reported to reduce neuronal loss, OS, and neuroinflammation in rodent models of PD and AD. These agents scavenge lipid radicals and maintain cell membrane integrity, thereby preventing ferroptosis-induced neurotoxicity [203,204]. Furthermore, iron chelators such as deferiprone have been studied to reduce brain iron burden and attenuate disease progression [205,206].

Despite these promising findings, the clinical application of ferroptosis-targeted therapies in neurodegeneration remains in its early stages of development. Further research is needed to determine the temporal role of ferroptosis, identify biomarkers of susceptibility to its development, and elucidate its interactions with other forms of cell death in the aging brain. Current evidence suggests that ferroptosis is a common pathological mechanism in many neurodegenerative diseases and may represent a viable target for future neuroprotective therapies [199].

## 6. Analytical Tools in Ferroptosis Research

### 6.1. Lipidomics and Detection of Lipid Peroxidation Products

**Figure 3:**
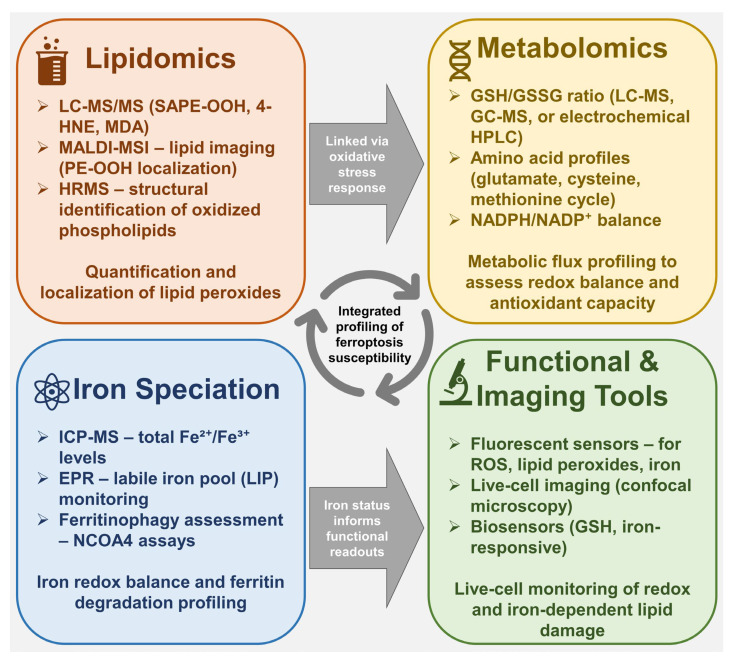
Analytical platforms supporting ferroptosis research through the profiling of redox status, lipid peroxidation, and iron metabolism. The figure summarizes four major methodological areas: (i) lipidomics—for detection of peroxidized lipid species and structural characterization, (ii) metabolomics—for measurement of antioxidant status (e.g., GSH/GSSG, NADPH/NADP^+^) and metabolic flux, (iii) iron speciation—for quantitative and functional assessment of intracellular Fe^2+^/Fe^3+^ levels, and (iv) functional and imaging tools—for dynamic monitoring of ferroptosis in live-cell and in vivo systems. These techniques collectively enable integrated profiling of ferroptosis susceptibility across biological contexts. Abbreviations used: EPR—electron paramagnetic resonance, Fe^2+^/Fe^3+^—ferrous/ferric iron, GSH—glutathione, GSSG—oxidized glutathione, HRMS—high-resolution mass spectrometry, HPLC—high-performance liquid chromatography, ICP-MS—inductively coupled plasma mass spectrometry, LC-MS/MS—liquid chromatography–tandem mass spectrometry, LIP—labile iron pool, MALDI-MSI—matrix-assisted laser desorption/ionization mass spectrometry imaging, NADP^+^—nicotinamide adenine dinucleotide phosphate (oxidized), NADPH—nicotinamide adenine dinucleotide phosphate (reduced), NCOA4—nuclear receptor coactivator 4, PE-OOH—oxidized phosphatidylethanolamine.

Lipidomics has emerged as a critical analytical platform for investigating the biochemical underpinnings of ferroptosis, particularly by enabling the detection and quantification of lipid peroxidation products [207]. During ferroptosis, enzymatic and non-enzymatic oxidation of PUFAs incorporated into membrane phospholipids leads to the generation of PUFA-OOH and secondary reactive species. These oxidation products not only serve as hallmarks of ferroptotic activity but also play causal roles in membrane disruption and cell death signaling (see Figure 3) [208].

Among the most commonly measured end-products of lipid peroxidation are MDA, 4-HNE, and F2-isoprostanes. MDA is a low-molecular-weight aldehyde formed from the oxidative cleavage of PUFA chains, frequently used as a general oxidative stress biomarker [209]. 4-HNE, derived from ω-6 PUFAs such as linoleic and arachidonic acid, is highly reactive and can form covalent adducts with proteins and nucleic acids, influencing cell signaling and viability [210]. F2-isoprostanes, non-enzymatically generated from arachidonic acid via free radical mechanisms, are considered reliable indicators of lipid peroxidation in vivo due to their chemical stability and structural specificity [211].

The accurate detection of these lipid-derived products requires advanced analytical methodologies. Gas chromatography–mass spectrometry (GC–MS) and liquid chromatography–tandem mass spectrometry (LC–MS/MS) are the gold-standard techniques for both targeted and untargeted lipidomics [212]. GC–MS is particularly effective for volatile or derivatized compounds such as MDA and 4-HNE, while LC–MS/MS provides superior sensitivity and selectivity for complex lipid species in biological matrices [213]. These approaches enable not only quantitative profiling of oxidized lipid species but also structural elucidation, providing insights into the specific phospholipid substrates and oxidation pathways active during ferroptosis [25].

Beyond quantification of peroxidation end-products, modern lipidomic workflows allow for comprehensive mapping of oxidized phospholipid species, particularly those implicated in ferroptosis, such as peroxidized phosphatidylethanolamines (PE-OOH) [214]. These species can be selectively enriched and identified using LC–MS/MS with multiple reaction monitoring (MRM) or high-resolution mass spectrometry (HRMS) platforms. Specific oxidized lipid signatures, for example, 1-stearoyl-2-15-hydroperoxy-eicosatetraenoyl-sn-glycero-3-phosphoethanolamine (SAPE-OOH), have been validated as proximal markers of ferroptosis and correlate with GPX4 inhibition or ACSL4 overexpression in model systems [215].

Such lipidomic profiles are not merely descriptive but can be functionally informative. Correlating lipid oxidation patterns with ferroptotic sensitivity enables the stratification of cell types, tissues, or even patient-derived samples based on their ferroptosis-prone lipid landscape [216]. For instance, tumors with elevated levels of oxidizable PEs or increased ACSL4 expression tend to exhibit enhanced accumulation of PE-OOH upon GPX4 suppression, providing a predictive marker for ferroptosis-inducing therapy. This approach has been particularly valuable in drug screening, where changes in the oxidized lipidome can be used to assess the efficacy and selectivity of novel ferroptosis modulators [217].

In situ lipid imaging techniques such as matrix-assisted laser desorption/ionization mass spectrometry imaging (MALDI-MSI) have further expanded the capabilities of ferroptosis research [218]. These tools enable spatially resolved analysis of oxidized lipids within tissue sections, offering unprecedented insight into regional lipid peroxidation and its pathological consequences. For example, MALDI-based imaging has revealed ferroptosis-associated lipid signatures in models of ischemia-reperfusion injury and neurodegeneration, suggesting that localized ferroptotic processes may contribute to tissue-specific damage patterns [219].

Collectively, lipidomics provides a high-resolution lens through which the biochemical landscape of ferroptosis can be visualized and quantified. By capturing both terminal oxidation products, such as MDA and 4-HNE, as well as upstream intermediates, including peroxidized phospholipids, lipidomic analyses provide mechanistic and diagnostic insights into ferroptotic processes [220]. Moreover, the integration of lipidomic data with genetic, pharmacological, and phenotypic readouts enables a systems-level understanding of how ferroptosis unfolds in diverse biological settings.

As analytical technologies continue to evolve, lipidomics is expected to play a pivotal role in biomarker discovery, therapeutic stratification, and mechanistic validation of ferroptosis-targeted interventions. However, lipid peroxidation represents only one aspect of the ferroptotic cascade. To fully characterize this form of cell death, it is essential to monitor complementary metabolic pathways-including those regulating antioxidant defense, redox homeostasis, and iron speciation. These dimensions are addressed through metabolomics, the focus of the following section.

### 6.2. Metabolomics Approaches

Metabolomics has become an indispensable tool in ferroptosis research, offering a global and quantitative view of small-molecule fluxes that reflect cellular redox balance, antioxidant capacity, and iron homeostasis. In contrast to lipidomics, which focuses on oxidized lipids, metabolomics provides insight into the broader biochemical networks that enable or restrain ferroptotic death. This includes pathways governing glutathione metabolism, NADPH generation, amino acid turnover, and iron redox cycling (see Figure 3).

A central parameter in ferroptosis-oriented metabolomics is the GSH/GSSG ratio, which reflects antioxidant capacity and indirectly indicates GPX4-dependent redox control [221]. Targeted metabolomics using high-performance liquid chromatography (HPLC) coupled with electrochemical detection or LC–MS/MS enables precise quantification of this ratio in cells and tissues undergoing ferroptosis [222].

Another crucial component of ferroptosis-related metabolomic profiling is the speciation of intracellular iron, particularly the balance between the redox-inactive Fe^3+^ and catalytically active Fe^2+^ pools, as well as protein-bound pools such as ferritin [223]. Analytical techniques, such as inductively coupled plasma mass spectrometry (ICP-MS) and electron paramagnetic resonance (EPR) spectroscopy, enable the quantification and characterization of these iron species. EPR, for instance, can detect labile Fe^2+^ complexes and track dynamic changes in response to ferroptosis inducers or iron chelators, providing functional insight into the catalytic landscape that supports lipid peroxidation [224].

Additionally, untargeted metabolomics using ultra-high-resolution mass spectrometry and multivariate data analysis has uncovered novel metabolic signatures associated with ferroptosis. These include alterations in amino acid metabolism (e.g., cysteine, glutamate), the methionine cycle, and intermediates of the tricarboxylic acid cycle [225]. Such data not only deepens mechanistic understanding but also aids in identifying potential biomarkers for ferroptotic activity in preclinical and clinical samples. For example, elevated levels of cystine, glutamate, and oxidized NADPH derivatives have been reported in cells undergoing ferroptosis induced by system Xc^−^ inhibitors [26].

The power of metabolomics lies not only in identifying isolated metabolic changes but also in its ability to reveal network-level perturbations that reflect ferroptotic vulnerability or resistance. When combined with lipidomics, these approaches can yield a comprehensive map of ferroptosis-related metabolic states-from iron-catalyzed peroxidation of membrane lipids to glutathione depletion and redox collapse. Such integrative analyses are particularly useful for dissecting the temporal sequence of ferroptosis, identifying early metabolic triggers, and distinguishing ferroptosis from other forms of regulated cell death.

Advances in single-cell metabolomics, isotope tracing, and spatial metabolite imaging are expected to further refine our understanding of ferroptosis in heterogeneous tissue environments [226]. These technologies will allow researchers to capture ferroptotic processes at unprecedented resolution, for example, pinpointing metabolic outliers within tumors or tracking ferroptosis-associated shifts in immune cell metabolism during infection. Moreover, the development of real-time biosensors and non-invasive imaging probes targeting glutathione or labile iron may offer future clinical tools for monitoring ferroptotic stress in vivo.

Together, metabolomics and lipidomics form the analytical backbone of ferroptosis research, enabling the quantitative dissection of a process that is inherently metabolic, redox-driven, and context-dependent. These tools not only support mechanistic studies but also serve as a foundation for biomarker discovery and therapeutic stratification, moving ferroptosis from basic biochemical inquiry toward translational application.

## 7. Future Directions and Therapeutic Perspectives

The therapeutic application of ferroptosis modulation is rapidly evolving. Promising strategies are emerging to treat cancer and infections more effectively. Combination therapies appear to be a key part of this progress. The use of ferroptosis inducers in combination with chemotherapy or immunotherapy may be beneficial. For example, erastin or RSL3, inducers of ferroptosis, act synergistically with chemotherapeutic agents such as cisplatin. To overcome the drug resistance of aggressive tumors, they use the metabolic susceptibility of tumor cells [227,228]. Similarly, coupling ferroptosis inducers with immune checkpoint inhibitors (e.g., anti-PD-1/PD-L1) enhances antitumor immunity by promoting immunogenic cell death and activating dendritic cells [229,230]. This approach leverages the release of tumor-associated antigens and damage-associated molecular patterns to stimulate T-cell responses, thereby addressing the immunosuppressive tumor microenvironment [229,230].

Overcoming resistance mechanisms remains critical. Tumors often evade ferroptosis through the upregulation of NRF2-mediated antioxidant pathways or the ferroptosis regulator FSP1, which scavenges lipid peroxides [231,232]. Novel inhibitors targeting NRF2 (e.g., brusatol) or FSP1 (e.g., iFSP1) are being developed to sensitize resistant cancers [183]. Concurrently, infection-induced ferroptosis, observed in sepsis and COVID-19, presents unique therapeutic opportunities. In sepsis, pathogens trigger ferroptosis in immune cells, thereby exacerbating immunosuppression and organ failure [233]. Targeted inhibition of ferroptosis using liproxstatin-1 or deferoxamine reduces mortality by preserving the function of immune cells [188,234,235]. For COVID-19, ferroptosis contributes to pulmonary damage and the development of cytokine storms. Thus, interventions such as iron chelators or GSH precursors may mitigate severity [236].

There are still challenges in achieving selectivity and minimizing toxicity outside the target area of use. Ferroptosis modulators often lack tissue specificity, which can lead to damage to healthy cells [231]. To address this, nanoparticle-based delivery systems show promise. Lipid-based nanoparticles or metal-organic frameworks can encapsulate ferroptosis inducers or iron oxide nanoparticles, enabling tumor-specific targeting via enhanced permeability and retention effects or surface-functionalized ligands (e.g., folate receptors) [229]. These systems enhance pharmacokinetics and reduce systemic toxicity while amplifying lipid peroxidation in malignant cells. Additionally, synthetic ferroptosis modulators with tunable redox properties are being designed for precise spatiotemporal control [229,231].

Future tools to modulate ferroptosis regulators include advanced delivery forms such as stimuli-responsive nanoparticles and gene editing approaches (e.g., CRISPR-Cas9) [237]. However, clinical translation requires resolving tissue-specific biodistribution, long-term safety, and scalability issues. Integrating multi-omics profiling (lipidomics/metabolomics) will further refine patient stratification for ferroptosis-targeted therapies.

## 8. Conclusions

Ferroptosis represents a distinct and chemically defined form of regulated cell death driven by iron-dependent lipid peroxidation. Its execution relies on a complex interplay between redox-active iron, the incorporation of polyunsaturated fatty acids into membrane phospholipids, and the failure of antioxidant systems such as GPX4 and FSP1. The susceptibility of cells to ferroptosis is not a fixed trait but rather a dynamic outcome shaped by metabolic state, iron availability, lipid remodeling, and inflammatory cues. In pathological contexts, such as cancer and infection, these factors undergo profound reprogramming, creating windows of vulnerability that can be therapeutically exploited.

Advances in analytical platforms, particularly lipidomics and metabolomics, have expanded our ability to monitor ferroptotic processes with high resolution and contextual specificity. These tools not only support mechanistic understanding but also enable the identification of biomarkers and treatment targets. From now on, integrating ferroptosis profiling with cell-type-specific metabolic and inflammatory signatures will be critical for the development of selective ferroptosis-inducing or -protective strategies in disease.

Understanding the biochemical and metabolic logic of ferroptosis is essential not only for elucidating fundamental cell death mechanisms but also for harnessing this pathway in translational medicine. Whether by promoting ferroptosis in therapy-resistant tumors or mitigating its effects in infection-related tissue damage, targeted manipulation of ferroptosis holds promise for next-generation therapeutic approaches.

## Figures and Tables

**Table 1:** Summary of the mechanisms of action of key ferroptosis inducers.

Compound	Mechanisms of Action	Key Characteristics
Erastin	Inhibits the system Xc^−^ transporter, reducing cystine uptake and depleting GSH levels.	Enhances the uptake of chemotherapeutic drugs in resistant tumors by modulating iNOS.
RSL3	Directly binds and inhibits GPX4 via covalent modification of its active site.	Highly effective in tumors with elevated GPX4 expression (e.g., non-small cell lung cancer).
FIN56	Degrades GPX4 and activates squalene synthase, depleting CoQ10 antioxidant.	Dual-action: reduces GPX4 protein levels and increases oxidative stress.
FINO_2_	Indirectly disrupts GPX4 and oxidizes intracellular iron to generate ROS.	Contains endoperoxide/hydroxyl structure; selective for cancer cells (e.g., HT-1080, RS411).
Sulfasalazine	Inhibits system Xc^−^, depleting GSH.	FDA-approved for inflammatory diseases (e.g., rheumatoid arthritis); exhibits anti-cancer properties.
Sorafenib	Disrupts the system Xc^−^ function.	Oral multitarget kinase inhibitor used for thyroid, liver, and renal cell carcinomas.

Abbreviations: CoQ10—coenzyme Q10; FDA—Food and Drug Administration; GSH—glutathione; GPX4—Glutathione Peroxidase 4; iNOS—inducible nitric oxide synthase.

**Table 2:** Summary of the mechanisms of action of key ferroptosis inhibitors.

Compound	Mechanisms of Action	Key Characteristics
Liproxstatin-1	Radical-trapping antioxidant	Stops lipid peroxidation chain reactions; more effective in lipid membranes than in solution.
Ferrostatin-1	Radical-trapping antioxidant	Neutralizes lipid peroxyl radicals more efficiently than vitamin E.
Vitamin E	Lipophilic antioxidant	Neutralizes lipid radicals, but is weaker than synthetic RTAs.
Coenzyme Q10 (CoQ10)	Lipid-soluble antioxidant	FSP1-CoQ10-NADPH axis regenerates reduced CoQ10.
Deferoxamine (DFO)	Iron chelator	Binds free iron to block the Fenton reaction.
Deferiprone (DFP)	Iron chelator	Binds free iron to inhibit iron-dependent lipid peroxidation.

Abbreviations: CoQ10—Coenzyme Q10; FSP1—Ferroptosis Suppressor Protein 1; NADPH—Nicotinamide Adenine Dinucleotide Phosphate; RTAs—Radical-trapping antioxidants.

**Table 3:** Summary of the mechanisms of action of natural compounds targeting ferroptosis.

Compound Class	Compound	Mechanisms of Action	Key Effects
Polyphenols	Quercetin	↓ GPX4 & SLC7A11 expression ↑ NCOA4-dependent ferritinophagy ↑ Iron uptake	GSH depletion ↑ Lipid peroxidation ↑ ROS production
	EGCG	Suppresses GPX4 Blocks the NRF2 pathway ↓ Mitochondrial membrane potential	↑ Lipid peroxide accumulation Sensitizes cancer cells
	Curcumin	↓ SLC7A11 Activates ACSL4 Iron chelation	Lipid remodeling ↑ Fenton reactions ↑ OS
Terpenoids	Artemisinin derivatives	Iron-activated endoperoxide bridges ↓ Mevalonate pathway Inhibits GCLC	↑ Carbon-centered radicals ↓ CoQ10 production ↑ Lipid peroxidation
	Parthenolide	Inhibits GCLC	↓ GSH levels ↓ GPX4 activity
Flavonoids	Baicalein/Luteolin	↑ TFR1 expression ↓ Ferritin ↓ GPX4 Activates ACSL4	↑ Iron uptake ↑ PUFAs incorporation ↑ Membrane oxidation

Abbreviations: ACSL4—Acyl-CoA synthetase long-chain family member 4; CoQ10—Coenzyme Q10; GCLC—Glutamate-Cysteine Ligase Catalytic Subunit; GSH—glutathione; GPX4—Glutathione Peroxidase 4; NCOA4—Nuclear Receptor Coactivator 4; NRF2—Nuclear factor erythroid 2-related factor 2; OS—oxidative stress; PUFAs—Polyunsaturated Fatty Acids; ROS—Reactive oxygen species; SLC7A11—Solute Carrier Family 7 Member 11. Arrows indicate direction of change: ↑ denotes an increase, ↓ denotes a decrease.

## Data Availability

No new data were created or analyzed in this study. Data sharing is not applicable to this article.
